# Surgical treatment experiences in two cases of spontaneous esophageal ruptures

**DOI:** 10.1093/jscr/rjaf406

**Published:** 2025-06-09

**Authors:** Baoxiang Pei, Wenlong Gu, Qingmin Guan, Biao Kong

**Affiliations:** Department of Thoracic Surgery, Jining First People's Hospital, No. 6 Jiankang Road, Jining 272011, Shandong Province, China; Department of Thoracic Surgery, Qufu People's Hospital, No. 1 Guiren Street, Qufu, Jining City, Shandong Province, 272011, China; Department of Thoracic Surgery, Qufu People's Hospital, No. 1 Guiren Street, Qufu, Jining City, Shandong Province, 272011, China; Department of Thoracic Surgery, Qufu People's Hospital, No. 1 Guiren Street, Qufu, Jining City, Shandong Province, 272011, China

**Keywords:** spontaneous esophageal ruptures, surgical treatment, esophageal repair, late complications

## Abstract

Introduction and importance: Spontaneous esophageal ruptures (SREs), also known as Boerhaave syndrome, are rare but potentially fatal medical emergencies characterized by full-layer perforation of the esophagus. Early identification and surgical intervention are critical for improving patient outcomes and reducing mortality rates. Case presentation: This study presents two case reports of patients with SREs who underwent surgical treatment. Both patients experienced lower esophageal segment perforations and were treated with emergency surgery for primary esophageal repair. One patient developed severe septic shock postoperatively and required intensive care and conservative treatment. Both patients recovered completely and had no late complications, such as esophageal strictures or feeding tube issues, at 6-month follow-up. Clinical discussion: This study reports surgical treatment experiences in two cases of SRE, a rare and fatal condition characterized by full-layer perforation of the esophagus. Early identification, diagnosis, and prompt surgical intervention are crucial for improving patient survival chances. The two cases involved lower esophageal segment perforation and underwent emergency surgery for primary esophageal repair. Healthcare providers should be vigilant for Boerhaave syndrome in patients presenting with acute chest and abdominal pain, particularly those with a history of vomiting. Thorough irrigation of the thoracic cavity during surgery is key to preventing septic shock postoperatively. Conclusion: Early diagnosis and prompt surgical intervention are essential for managing SREs. Thorough surgical debridement and drainage are key to preventing postoperative complications and improving survival rates.

## Introduction

Spontaneous rupture of the esophagus (SRE), also known as Boerhaave syndrome, is a rare and fatal acute surgical emergency characterized by full-layer perforation of the esophagus, which was first described by Hermann Boerhaave in 1724 [1]. This condition is mostly caused by a sudden rise in esophageal pressure after overeating and excessive drinking. [2]. After esophageal rupture and perforation, gastric digestive juices and esophageal contents extravasate into the mediastinum pleural space, causing mediastinal and thoracic infections leading to septic shock and sepsis, with rapid progression of the disease, high misdiagnosis and mortality, and it is easily confused with acute myocardial infarction, gastroduodenal perforation, acute pancreatitis, and other cardiac and emergencies, with a mortality rate of over 40% [2]. Early recognition, diagnosis, and prompt surgical treatment can effectively improve the survival chances of patients. This article presents a retrospective analysis of clinical data and management procedures for two patients who were treated for spontaneous esophageal rupture (SRE).

## Case report

**Figure 1:**
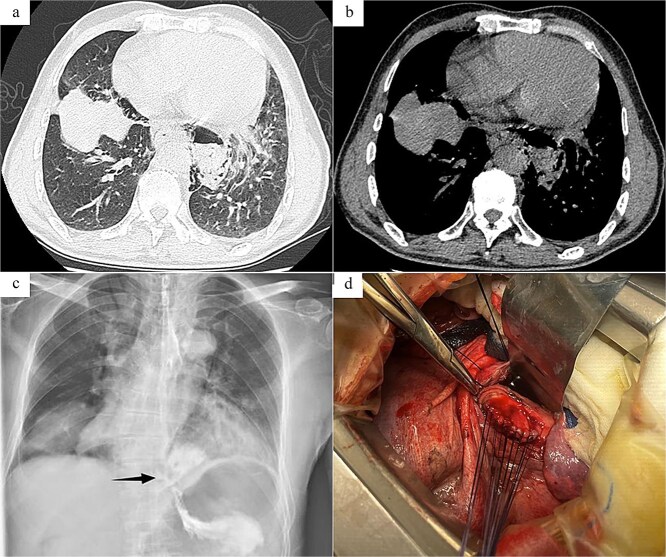
Radiology images and emergency surgery photos of spontaneous esophageal rupture (Case 1). (a and b) Chest and abdomen computed tomography revealed mediastinal emphysema around the lower segment of the esophagus. (c) Esophagus echoendoscopy showed contrast agent leakage into the perihilar region (arrow). (d) Emergency surgery revealed a rupture site located in the lower segment of the esophagus, with a length of about 4.0 cm, and involved interrupted absorbable sutures for full-layer closure of the esophageal perforation.

Case 1: The patient, a 67-year-old male, was admitted to Emergency Department due to “hematemesis for 2 h, abdominal and thoracic pain for 1 h.” On admission, imaging studies including chest and abdomen computed tomography (CT) revealed esophageal distal middle-third wall thickening, perihilar pleural edema, pericardial effusion (Fig. 1a and b). Esophagus echoendoscopy showed contrast agent leakage into the perihilar region (Fig. 1c). Electrocardiogram showed no abnormalities. Blood routine test showed that white cell count 11.7 × 10^9^/L, neutrophil count 10.4 × 10^9^/L, neutrophil percentage 89%. In summary, the diagnosis was SRE. In emergent surgery under general anesthesia, laparoscopic repair of esophageal tear was performed. Intraoperative exploration revealed a rupture site located in the lower segment of the esophagus, with a length of about 4.0 cm (Fig. 1d), esophagus and gastric contents (stomach) overflowed at the site of the injury. The surgical steps included removal of foreign bodies, copious irrigation with normal saline until clear, and iodine solution irrigation for disinfection. Subsequently, the surgery trimmed the severely contaminated mucosa and muscular layer of the esophageal perforation until slight oozing of blood, involved interrupted absorbable sutures for full-layer closure of the esophageal perforation, reflushed with iodine solution. Drainage tubes were placed in the mediastinum and chest cavity, respectively. And a gastric tube and a duodenal feeding tube were indwelled. Postoperative care included anti-infection, protective measures, and parenteral nutrition. On the second day after surgery, the patient developed febrile maintained at 38.1°C–38.6°C, and a repeat blood routine examination showed low white blood cell count. The heart rate remained around 125 beats per minute, the blood pressure fluctuated between 80–90/50–60 mmHg, with palpitations, apathy, and involuntary spasms in the limbs. Infectious shock was considered after multi-disciplinary discussion, and the patient was transferred to the intensive care unit (ICU) for upgraded antibiotics (imipenem, vancomycin) therapy, and was given ventilator-assisted breathing, fluid infusion, blood transfusion, enteral nutrition, and other supportive treatment. After 7 days of treatment in the ICU, the septic shock was corrected and the patient was transferred back to the general ward for treatment. Subsequently, the patient was reexamined with upper gastrointestinal radiography and chest CT, which suggested that there was no leakage or stenosis at anastomosis of the esophagus. After a total of 18 days of fasting, the patient began to resume a diet and was discharged in good health.

**Figure 2:**
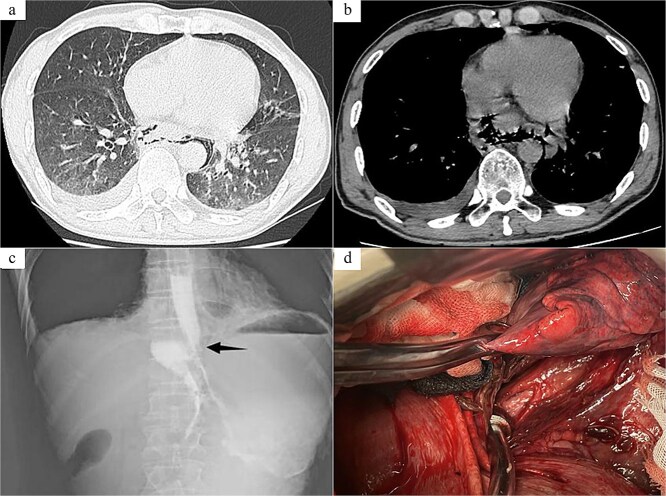
Radiology images and emergency surgery photos of spontaneous esophageal rupture (Case 2). (a and b) Chest and abdomen computed tomography revealed periesophageal air around the lower segment of the esophagus, mediastinal emphysema, multiple air in abdominal cavity, bilateral pleural effusion. (c) Esophagus echoendoscopy showed contrast agent leakage at the distal aboral esophageal segment (black arrow). (d) Emergency surgery revealed a long tear of about 3.0 cm in the posterior wall of the lower esophagus near the cardia.

Case 2: The patient, a 54-year-old male, was admitted to Emergency Department due to “nausea, hematemesis accompanied by abdominal and thoracic pain for half a day.” On admission, imaging studies including chest and abdomen CT revealed periesophageal air around the lower segment of the esophagus, mediastinal emphysema, and multiple air in abdominal cavity, bilateral pleural effusion (Fig. 2a and b). Esophagus echoendoscopy showed contrast agent leakage at the distal aboral esophageal segment (Fig. 2c). Electrocardiogram showed no abnormalities. Blood routine test showed that white cell count 12.49 × 10^9^/L, neutrophil count 11.19 × 10^9^/L, neutrophil percentage 89.6%. In summary, the diagnosis was SRE. In emergent surgery under general anesthesia, laparoscopic repair of esophageal tear was performed. Intraoperative exploration revealed a long tear of about 3.0 cm in the posterior wall of the lower esophagus near the cardia (Fig. 2d), and the esophageal wall and mediastinal pleura around the tear were observed to have grayish-brown-like changes, and a long interspace about 5.0 cm was seen under the post-cardiac abdominal membrane, which was filled with gastric contents that had overflowed. The same surgical approach as Case 1 was employed. The surgical method was the same as Case 1. And a T-shaped tube was placed in the subphrenic space behind the cardia for drainage and drainage tubes were placed in the posterior mediastinum and the chest cavity, respectively. Postoperative anti-infection, gastrointestinal protection, and enteral nutrition therapy were given. Half a month after the operation, the patient was examined with upper digestive tract radiography and chest CT, which showed that there was no leakage or stenosis the anastomotic site of the esophagus. The patient was allowed to resume eating after fasting for 16 days and was discharged after recovery.

Both patients experienced spontaneous recovery of infection-induced esophageal injury and had no late complications such as anastomotic stenosis or reflux esophagitis. Both patients had given informed consent for the publication of case report. The work has been reported in line with the Surgical Case Report criteria [3].

## Discussion

Esophageal perforation is a serious gastrointestinal emergency that can lead to severe complications if not treated promptly. There are many causes of esophageal perforation, with the most common being iatrogenic perforations, such as those occurring during upper gastrointestinal endoscopy, esophageal dilation, and transesophageal echocardiography [4]. SRE is very rare among esophageal perforations, with an estimated incidence of about 3.1 cases per million people per year, accounting for 15% of all esophageal perforations, primarily affecting individuals aged 40 to 60 years, with 80% being male [5, 6]. The main symptoms of SRE include severe vomiting, chest pain, and subcutaneous emphysema, known as Boerhaave syndrome [1]. The pathophysiology is based on a sudden increase in intraluminal esophageal pressure and intrathoracic negative pressure, but not all patients exhibit this triad of symptoms [1]. In many cases, the clinical presentation is nonspecific and can easily be confused with other conditions causing chest and abdominal pain, such as gastric ulcer perforation, myocardial infarction, pulmonary embolism, aortic dissection, and pancreatitis, leading to a high risk of misdiagnosis. Consequently, many patients are either diagnosed late or completely misdiagnosed. After esophageal rupture, digestive fluids leak into the thoracic cavity, quickly causing surrounding infections, mediastinitis, widespread infection in the thoracic cavity leading to septic shock, and high mortality rates, especially when diagnosed late (after 24 h), where mortality can reach 40% [2]. In most cases, the rupture occurs in the lower esophagus, ~2–4 cm from the gastroesophageal junction, where the esophageal wall is relatively weak, with left-sided wall ruptures being more common than right-sided ones [7].

The prognosis of SRE largely depends on the speed of diagnosis, requiring a comprehensive assessment, including a careful medical history and diagnostic methods. Endoscopy has high sensitivity for detecting and showing the extent of the rupture, but caution is necessary as it may exacerbate the tear and worsen the patient’s clinical condition. Chest and abdominal CT can differentiate and rule out emergencies such as gastrointestinal perforation and acute pancreatitis, with CT being highly sensitive to gas in the mediastinum, making it easy for mediastinal emphysema caused by esophageal rupture to spread within the mediastinal spaces. Early esophagography can help accurately diagnose this rare condition [8]. In the first case presented in this article, the patient was suspected of having a gastrointestinal emergency, and an upper abdominal CT indicated thickening of the lower esophageal wall and mediastinal emphysema. Further chest CT showed significant mediastinal emphysema and a small amount of left pleural effusion, highly suspecting esophageal rupture, which was confirmed by esophagography. The second patient presented slightly later, and based on the diagnostic experience from Case 1, the attending physician promptly completed a chest and abdominal CT, which indicated thickening of the lower esophageal wall, mediastinal emphysema, and bilateral pleural effusion, leading to a direct esophagography diagnosis of SRE.

Treatment for SRE is divided into conservative and surgical approaches. Conservative treatment is suitable for patients with stable conditions, early presentation (<24 h), and no contamination of surrounding spaces, with a possibility of spontaneous healing of the perforation. However, close monitoring is required during treatment, and if symptoms progressively worse, surgical intervention is necessary [4, 9]. Most studies still regard surgical intervention as the gold standard, especially since this condition is relatively rare in thoracic surgery [10–12]. Early surgical treatment can improve patient survival rates, with the principles of management being to close the rupture as soon as possible, control infection, and ensure adequate drainage. Common surgical types used clinically include simple esophageal repair, one-stage esophagectomy, esophageal stent placement, and thoracoscopic-assisted esophageal repair [11]. In the early stages of perforation, generally within 24 h, surgical exploration of the thoracic cavity typically reveals a small rupture, and in cases of mild infection, one-stage direct suture repair is usually performed. For patients diagnosed after 24 h with severe thoracic infection and poor overall condition, esophageal stent placement or combined thoracoscopic surgery may be considered [13, 14]. Ruptures in the upper and middle esophagus are usually treated via the right thorax, while lower esophageal perforations are more effectively approached through the left thorax [12]. In the two cases of esophageal rupture presented in this article, both patients sought medical attention within 24 h, with ruptures located in the lower esophagus, measuring 4–5 cm in length, and underwent one-stage surgical repair through left thoracotomy. Postoperatively, Case 1 developed septic shock during treatment but was cured and discharged after anti-infection therapy. Through the treatment process of these two patients, this article emphasizes that thorough irrigation of the thoracic cavity during surgery is key to preventing septic shock postoperatively.

## Conclusion

Increasing healthcare personnel’s awareness of SRE, early identification and diagnosis, and prompt surgical treatment are crucial for managing this condition. Clinicians on the front line should be vigilant for the possibility of Boerhaave syndrome in patients with acute chest and abdominal pain and a history of vomiting, selecting appropriate diagnostic methods to reduce misdiagnosis, and mortality rates.
